# Epidemiology and symptomatology of depression in Sri Lanka: A cross-sectional population-based survey in Colombo District

**DOI:** 10.1016/j.jad.2009.08.014

**Published:** 2010-06

**Authors:** Harriet A. Ball, Sisira H. Siribaddana, Yulia Kovas, Nick Glozier, Peter McGuffin, Athula Sumathipala, Matthew Hotopf

**Affiliations:** a MRC Social Genetic and Developmental Psychiatry Centre, Institute of Psychiatry, King's College London, De Crespigny Park, London, SE5 8AF, UK; b Sri Lanka Twin Registry, Institute of Research and Development, Battaramulla, Sri Lanka; c Sydney Medical School, University of Sydney, Sydney, Australia; d Department of Psychological Medicine, Institute of Psychiatry, King's College London, UK; e Section of Epidemiology, Institute of Psychiatry, King's College London, UK

**Keywords:** Depression, Epidemiology, Sri Lanka, Environment, Risk factors

## Abstract

**Background:**

It is important to understand the nature of depression in non-Western and lower-income countries, but little such research exists. This study aimed to examine the characteristic features of depression in Sri Lanka, and to identify environmental risk factors.

**Methods:**

Depression diagnoses, symptoms and impairment were measured using the Composite International Diagnostic Interview, in a population-based sample of 6014 twins and non-twins in the Colombo region of Sri Lanka (the CoTASS sample). Socio-demographic factors and environments were assessed via questionnaires.

**Results:**

Lifetime-ever depression was reported in 6.6% of participants, rising to 11.2% if the functional impairment criterion was excluded. The symptom profile of depression and its socio-demographic associations were very comparable to those in Western and more economically developed countries, whether functional impairment was included in the definition or not. Standard of living was independently associated with depression, especially among men at the more deprived end of the distribution. Specific associations were found with both financial wellbeing and material characteristics of the home environment.

**Limitations:**

The observational associations identified are cross-sectional, so do not necessarily imply causal links.

**Conclusions:**

Aside from a lower prevalence, depression is very similar in this predominantly urban Sri Lankan sample to higher-income, Western countries, and may be under-identified due to a relatively low cultural appropriateness of the assessment of impairment. Under Sri Lanka's cultural and environmental context, certain aspects of the material environment are associated with depression among certain segments of society, perhaps because of their particular link to social status and social networks.

## 1 Introduction

An increasing body of international epidemiological studies has shown that depression is common in most countries (Murray and Lopez, 1996), but that prevalence estimates vary considerably, and much more so than they do for rarer disorders such as schizophrenia and bipolar affective disorder (Weissman et al., 1996; Simon et al., 2002; The WHO World Mental Health Survey Consortium, 2004). Many potential factors might explain this variation.

Firstly, there has, for many years, been a debate about the cultural validity of international comparisons. It has been argued that symptoms such as low mood are culturally determined and may have little salience in some countries (Summerfield, 2006, 2007). A counter-argument is that the core symptoms of depression can be elicited and are readily understood in different populations (Patel, 2001), and that there is remarkable similarity in the pattern of symptoms of depression no matter where the disorder is measured (Simon et al., 2002). There is an urgent need to study common mental disorders in developing countries, where the majority of the world's population lives but very little research is conducted (Sumathipala et al., 2004; Sharan et al., 2007), and to use such research to influence health and social policy (Patel, 2001).

A second reason why prevalence of depression might fluctuate is that some relatively specific aspects of definition and measurement do not transplant between healthcare systems — so for example some diagnostic algorithms require depressive symptoms of a severity to lead the participant to seek medical help — which may not be an option in some places (Hicks, 2002; Goldney et al., 2004). Thirdly, variations may be an artefact of study design, if the survey took place in a particularly economically deprived centre, or in a health care setting (Simon et al., 2002). Finally, differences in risk factor profiles might explain variations. In the WHO Consortium Survey (The WHO World Mental Health Survey Consortium, 2004), Asian and African countries (China, Japan and Nigeria) had low prevalence compared to most other centres mainly in Europe and North and South America. This might, for example, be a result of genetic differences between populations, or differences in socio-cultural milieu, or a reporting bias.

Despite the variation in prevalence, international studies show that within individual populations several important associations have been replicated (Patel et al., 1999). Depression appears universally more common in women, the less well educated, the divorced or widowed, and the poor.

In this paper we describe a population-based study of depression in Colombo, Sri Lanka. No previous population-based studies have been reported from Sri Lanka, where depression epidemiology has particular public health salience due to the high suicide rate (Eddleston et al., 1998), and the impact of a prolonged civil war. Genetic analyses on data from the same sample (Ball et al., 2009) found sex differences in heritability patterns, with depression showing low heritability in men but high heritability in women. In the present paper, as well as describing the epidemiology of depression, we explore potential interactions between key environmental risk factors and gender. Our aims then were: (i)to describe the prevalence of depression according to different definitions; (ii)to describe the nature of depressive symptoms among individuals diagnosed with depression; (iii)to describe associations between gender, education and poverty on depression and (iv)to explore interactions between gender and other risk factors.

## 2 Methods

The study received approvals from the Institute of Psychiatry, King's College London Research Ethics Committee; the Ethical Review Committee, University of Sri Jayewardanepura; and the World Health Organisation's Research Ethics Committee.

### 2.1 Study design and participants

This was a population-based twin study with a comparable non-twin sample, the Colombo Twin And Singleton Study (CoTASS). Full details of the design and implementation of the study are described elsewhere (Siribaddana et al., 2008). Briefly, the study took place in the Colombo District of Sri Lanka, an area with a population of 2.2 M which includes the island's capital. The District has a mixture of urban and rural populations with 45% of the population officially designated as living in rural communities (Department of Census and Statistics - Sri Lanka, 2001). This area was selected in part due to its diverse population, which may include people who are more Westernised than in other areas of Sri Lanka; includes extremes of wealth and poverty; and attracts economic migrants as well as individuals displaced by the Sri Lankan Civil War and the tsunami.

The annual update of the electoral register consists of a household census. We added a question asking whether the householder knew of any twins, and identified 19,302 individual twins by this method. Of these, we randomly selected 4387 twins to take part in the project on common mental disorders. 4024 (91.7%) participated, and interviews were completed for 3995 (including 72 unpaired twins and 5 sets of triplets). Of the 1954 complete pairs, 1420 were same-sex pairs (of which 635 were male–male, and 830 were classified as monozygotic), and 534 were opposite-sex pairs. In addition, we conducted a parallel study of non-twins, randomly sampled from the same local areas from which twins were recruited. 2311 non-twins were selected and eligible to participate, of whom 2019 (87.4%) consented and were interviewed. The twin and non-twin samples had similar age and sex profiles (Siribaddana et al., 2008). We included all consenting individuals aged 15 years or older who spoke sufficient Sinhala to understand the interview. Individuals were excluded if they failed a mini mental state examination, or where interviews were conducted via a proxy.

The 3995 twins included 3600 of Sinhala ethnicity (90%), 230 Moors (5.8%), 147 Tamils (3.7%) and 18 people from other ethnic groupings (including Burghers) (0.5%). The rate of not giving consent was slightly higher among non-Sinhala than Sinhala twins (12.9% versus 8.7%, *z* = 2.94, *P* < 0.01) but this did not differ between the two largest non-Sinhala groups (Tamils and Moors, *z* = 0.30, *P* = 0.76). Slightly more of those excluded due to non-fluency in Sinhala were Moors rather than Tamils (59 versus 26, out of the initial random sample of 6600 twins), but this probably reflects the higher rate of Moors encountered in the study. Due to power constraints, analyses based on ethnicity have collapsed participants into Sinhala and non-Sinhala categories, with further analyses to explore any differences found.

Interviews took place between 2006 and 2007, when Sri Lanka had been experiencing violent civil war for over 20 years. A small minority (2.6%) of the participants reported directly participating in the conflict as combatants. There have been uprisings and bombing attacks in Colombo, and at times a strong military presence. However this region has not been as heavily involved as have areas in the north and east of the island. Similarly, while many people in Colombo have been affected by the tsunami of 2004, direct involvement was not on the same scale as in the south of the island.

### 2.2 Data collection

Research workers visited the participants' homes to interview them each separately. We used the Composite International Diagnostic Interview (World Health Organisation, 1990), as it is a structured diagnostic interview for use by lay interviewers. We used qualitative techniques to adapt the measures. We sent the measures to a total of 13 bilingual twins (contacted from the registry) and other Sri Lankans fluent in English and Sinhala. Each measure (or in some cases subcomponent of measures) was translated at least twice independently. The translations were then reviewed in group meetings consisting of 7 professionals (6 doctors and 1 health service researcher, all with a background relevant to the measures — e.g., mental health) over a period of 4 months. A scholar in Sinhala also checked the translation. The adaptation was not a direct, literal translation, but aimed to find forms of words in Sinhala that best described the concepts of interest and where the questions when translated seemed cumbersome, they might be broken down into two component items for clarity. The interviews were then trialled by multiple volunteers recruited from field workers and four individuals with no connection to the study, in order to confirm that lay people could understand it.

For present analyses we defined three categories of depression (not mutually exclusive).

(1) Conventional DSM-IV criteria for a depressive episode (World Health Organisation, 1990) (ignoring bereavement criterion, and opt-out due to mixed states) (‘DSM’)
(2) A less rigorous definition which met the DSM criteria apart from the requirement for reported functional impairment (‘DSM excluding impairment’)
(3) Scoring positively on at least one ‘probe’ question (a two-week period of either (i) feeling sad, empty or depressed; or (ii) loss of interest in most things) (‘D-probe’).

All three categories were assessed as lifetime-ever experiences. In addition, those with DSM diagnoses were further probed to assess presence of the disorder currently or in the past year.

A questionnaire assessed socio-demographic characteristics and current living environment based on items from the Sri Lanka census. Items tapped into a wide spectrum of household characteristics rather than just detecting the poorest end of the distribution. Items included: house tenure and type (3 items); overcrowding (1 item); quality of structural materials (3 items); toilet and water facilities (3 items); lighting and fuel type (2 items); household commodities (4 items); access to means of transport (1 item); a subjective report of one's financial situation (1 item); and experiencing hunger due to poverty in the last three months (1 item). A composite standard of living variable (‘SoL’) was created by summing these items; the alpha reliability was 0.81.

### 2.3 Statistical analysis

A database combining the twin and non-twin data was constructed in SPSS (SPSS, 2005). Analyses were performed in Stata v9.2 (Stata, 2007). Analyses were corrected for the non-independence of twins within pairs, using the Huber–White–Sandwich (robust) estimator of variance. Genetic analyses of the depressive phenotypes that make use of the zygosity information are reported elsewhere (Ball et al., 2009).

#### 2.3.1 Descriptive statistics

A prevalence of the definitions of depression described above was calculated on the whole sample, and stratified by socio-demographic characteristics. Odds ratios adjusted for all socio-demographic characteristics were calculated for the ‘DSM excluding impairment’ definition.

#### 2.3.2 Symptom profiles

Among the individuals meeting each definition of depression, we examined endorsement frequency for each of the 14 symptom items that make up the nine symptom groups recognised in DSM-IV criteria for depression. Regression models were used to test associations between symptoms reported and ‘impairment’ status, and sex.

#### 2.3.3 Standard of living

We examined the association of standard of living with depression (‘DSM excluding impairment’ definition) in a logistic regression model using the SoL composite, and also by comparing quintile groups of the SoL composite. This revealed a non-linear effect (poor standard of living predicted depression, but only below a certain level, among men). Therefore we divided the sample into four groups (stratified by sex, and high/low SoL, cut-off at the 40th percentile). Within these groups we examined whether people in certain social roles (marital status; employment in the past year: none/1–10 months/11–12 months) were more susceptible to depression in poor standards of living.

Finally, we describe associations between depression and specific components of poor standard of living, using logistic regression models and testing for interactions according to sex. These were: i) informal housing tenure/type (e.g., row house; or neither owned/rented by occupant), ii) overcrowding (two or more persons per room), iii) informal structural materials (e.g., metal sheet), iv) poor toilet or water facilities (e.g., pit latrine toilet, toilet/drinking water source shared across households), v) low quality lighting or fuel type (e.g., kerosene or fire wood), vi) low access to commodities: (no refrigerator/phone in the household), vii) low access to transport (nobody in the household has a motorised vehicle), viii) hunger due to poverty in the past three months, and ix) financial difficulty: participant reported finding it “difficult”/“very difficult” to make ends meet. In these final models, although the effects were independent of socio-demographic variables, each standard of living component was entered separately to avoid collinearity.

## 3 Results

### 3.1 Prevalence

Lifetime depressive episodes (‘DSM’) were experienced by 4.8% of men and 8.1% of women; of these participants, 18.9% had had more than one episode. 1.6% were currently experiencing a DSM episode, and 2.7% had experienced such an episode over the past year. Lifetime episodes that met ‘DSM excluding impairment’ criteria were experienced by 8.0% of men and 13.9% of women. A two-week period of feeling depressed or loss of interest (‘D-probe’) was experienced by 13.4% of men and 17.9% of women (Table 1).

### 3.2 Socio-demographic factors

The socio-demographic characteristics of the participants meeting the three definitions of depression are presented in Table 1. Female sex, middle/old age, Sinhala ethnicity, being previously married and having been at school for less than ten years were all associated with depression regardless of the lifetime-ever definition used, and the same trends were seen in those with current or past year DSM depression, although the broader definitions lent more statistical power to test associations. Twin/non-twin status was not associated with depression. There was no main effect of living in an urban rather than a semi-urban area of Colombo, however this covered up an interaction effect with sex (*z* = 2.65, *P* = 0.008) for ‘DSM excluding impairment’: urbanicity was associated with depression in men but not in women.

Adjusted odds ratios for the ‘DSM excluding impairment’ definition of depression confirmed that these factors were independently associated with depression, and all were included as potential confounders when examining environmental associations below (although age was entered in years rather than as quartile categories when included as a confounder to avoid over-aggregation). Never married status was associated with lower rates of depression, but this association was not retained once controlling for the other factors, suggesting confounding by age (just 8% of participants in their 40 s had never been married). We further explored the finding of lower depression among the ‘non-Sinhala’ group, and found that it only held among the Moor group (OR = 0.42, *P* < 0.01).

### 3.3 Symptom profiles

Among participants who had had a lifetime-ever DSM episode of depression, an average of 7.0 symptoms were endorsed. The percentage who endorsed items one (depressed mood) and two (loss of interest) was very high (94% and 91%) because at least one of these symptoms is required by definition (Table 2). Changes in appetite or weight were common, but this was almost entirely accounted for by decreases rather than increases. The most frequently endorsed other items were insomnia, lack of energy and lack of concentration; over half of those affected reported thoughts of death. Psychomotor symptoms were relatively common (present in over half of cases), but the ‘guilt’ item was rare (just 20%). Similar patterns were seen when the affected group was restricted to those who currently or in the past year met a DSM definition.

The percentage endorsement of each symptom was lower when the requirement for impairment was removed. However, there was no evidence of a qualitative difference: the reduction in endorsement was at most seven percentage points, rather than certain items marking out those people who were in the full DSM definition group. This was tested using two regression models on the participants who qualified on at least the ‘DSM excluding impairment’ definition. First, impairment status was associated with symptom score (OR = 1.60, 95% CI 1.39–1.85). Second, impairment status was associated with nine variables representing the difference between total symptom score and each of the nine symptoms (i.e. the incremental effect of each symptom). In this second model, the coefficients relating to each symptom could be equated (Wald test of Eq. of parameters: *χ*^2^ = 8.10 for 8df, *P* = 0.42), indicating that although total symptom score was higher in impaired participants, there was no systematic elevation of particular symptoms. Similarly, the ‘D-probe’ group compared to the ‘DSM excluding impairment’ group had fewer endorsements for each item, and this reduction appeared uniform across different symptoms.

The symptom profile for the ‘DSM excluding impairment’ definition was further analysed by sex, which revealed sex-invariance for all but two items (at *P* < 0.01), and the differences were not large. Depressed men experienced significantly more hypersomnia (29%, versus 17% in women), and depressed women experienced significantly more thoughts about death (57%, versus 44% in men).

### 3.4 Standard of living

**Fig. 1:**
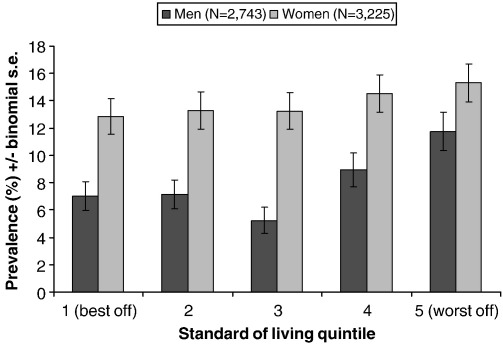
Prevalence of depression (‘DSM excluding impairment’ definition) according to sex and standard of living quintile.

The composite standard of living (SoL) variable was associated with depression (‘DSM excluding impairment’ definition) (Table 3). However, there was a significant interaction with sex (*z* = −2.24, *P* = 0.049), and when stratified by sex, the association was only seen in males. By splitting the whole sample into quintiles based on SoL score, it was observed that the bottom two quintiles (40th percentile and below) were more likely to be depressed, but there was little evidence of a dose–response relationship (Fig. 1). Again this effect was specific to males (OR = 1.76, 95% CI 1.30 – 2.36 for men in the bottom 40% compared to the top 60%, Table 3).

In order to investigate why low levels of standard of living appeared to be associated with depression among men only, we divided the sample into four groups according to sex and high/low SoL (40th percentile cut point) (Table 4). This revealed that being previously married (i.e. being a widow(er), separated or divorced) was a risk factor for depression among all four sex-by-SoL groups. However, working for some but not all months of the past year was a risk factor only in men of low SoL (adjusted OR: 1.71 (1.07–2.75), employment for 1–10 months compared to 11–12 months). In contrast, only among women of low SoL, those who did not work at all were significantly less depressed (adjusted OR: 0.59 (0.42–0.82) employment for 0 months compared to 11–12 months). No effects were seen in the high SoL groups, so it seems that the association of work pattern with depression is moderated by standard of living.

Finally, we examined the effect of binary risk components that contributed to the lower end of the SoL composite. We found that, after adjusting for socio-demographic variables, participants' reports of financial wellbeing were associated with depression regardless of sex (Table 3). However, poor quality structural materials, poor access to toilet or water facilities, and recent hunger due to poverty were all associated with depression only among men.

## 4 Discussion

The prevalence of lifetime-ever DSM-IV depressive episodes was found to be considerably lower in the current Sri Lankan sample (6.6%) than the same DSM-IV definition from the same diagnostic interview (CIDI) in Western countries (e.g., 12.8% in Europe (Alonso et al., 2004) and 16.6% in the USA (Kessler et al., 2005)). However, this finding is consistent with other Asian countries, for whom mood disorders are less common than in Europe and the Americas (e.g., 12 month prevalence was 3.1% in Japan and 2.5% in Beijing, compared to 2.7% in the current study) (Simon et al., 2002; The WHO World Mental Health Survey Consortium, 2004). However, we found that the socio-demographic correlates and symptom composition of depression in the current sample are broadly similar to previous reports from more developed countries. We also report novel associations of depression with certain aspects of standard of living, most saliently among underemployed men.

The current results are not necessarily generalisable to the whole island of Sri Lanka, in particular because we only sampled in one District that includes the capital city and neighbouring areas, and because the sample was predominantly of Sinhala ethnicity. Nonetheless, we gained a participation rate of over 90% and this is the first large epidemiological study of depression in Sri Lanka.

### 4.1 Symptoms of depression in the Sri Lankan sample

The generally similar pattern of symptom profiles to that from other countries (total symptom counts were similar; the same items were most frequent; slightly over half of those affected experience thoughts of death) (Kornstein et al., 2000; Middeldorp et al., 2006) suggests that we are studying the same disorder as seen in Western populations. This is consistent with research suggesting that different prevalences in different cultures are not due to ‘category fallacy’ or different validity or nature of the disorder in different countries (Simon et al., 2002). However, there were minor differences to previous reports from Western countries (Kornstein et al., 2000; Middeldorp et al., 2006). Psychomotor symptoms were relatively more common in the current Sri Lankan sample. The overall endorsement of appetite or weight changes was similar to other countries, but was heavily concentrated among reports of *loss* of appetite or weight in Sri Lanka. This may be connected to the Sri Lankan cultural perception of weight loss as unhealthy, relative to perceptions in the West. This pattern is also consistent with a greater representation of typical, rather than generally less severe atypical depression (Kendler et al., 1996) in this population. Also, the rarity of the ‘guilt’ item is perhaps due to cultural influences (e.g., the predominant Buddhist traditions found in Sri Lanka, rather than Judeo–Christian traditions more commonly found in Western countries) on either the concept of guilt, or the situations in which it is experienced and reported, making it less common across the Sri Lankan population, not just those who are depressed.

When we removed the ‘functional impairment’ requirement from the definition of depression, the symptom count remained high and the pattern of symptoms was similar (e.g., total symptom count dropped from 7.0 to 6.7; that found using DSM criteria in an Australian sample was in between these figures (Middeldorp et al., 2006)). This supports the use of such a definition as producing a very close disorder to one in which ‘impairment’ is required.

There is a small body of evidence examining differences in symptom profiles between men and women, which suggests more similarity than difference, and few sex differences have been found consistently (Kornstein et al., 2000; Khan et al., 2002; Middeldorp et al., 2006). The current study found only two sex differences in symptom profiles, and these were minor, in line with the previous findings.

### 4.2 Definitions of depression

These findings suggest that a definition of depression without a functional impairment requirement in the Sri Lankan context studied results in a disorder very similar (in terms of symptoms endorsed, and socio-demographic correlates) to full DSM diagnoses in the Western world. Past studies suggest that using identical diagnostic criteria in locations with a low prevalence identifies patients with more severe illness and impairment (when impairment was measured more comprehensively than in the CIDI) (Simon et al., 2002). For example, DSM symptoms have higher diagnostic thresholds for MDD in Korea compared to the USA, so the same symptoms occur (or are reported) only once the disorder has become more severe (Chang et al., 2008). Insisting on ‘impairment’ disqualified almost half of the participants in the current study who meet the necessary symptom requirements, even though the kinds of impairment necessary are perhaps more appropriate to depressed people in North American or European populations (Hicks, 2002). For example, depressed people in Sri Lanka may not see it as appropriate to seek help (medical or otherwise), take some form of treatment, or have the option to take time off work, for depressive symptoms. Thus, international comparisons may benefit from using this more inclusive definition.

The present analyses also lend weight to screening tools that consist of a positive response to ‘probe’ questions in structured interviews. Whilst not indexing a disorder, D-probe does appear to be a less extreme version of the same phenotype that includes depressive disorders. This lends weight to dimensional theories that suggest disorders are the quantitative extreme of a continuum throughout the population (Plomin et al., 2008). This is of relevance for quantitative genetic analyses, which require large numbers of affected individuals in order to resolve aetiological patterns (Neale et al., 1994).

### 4.3 Environmental associations

We used a standard of living composite that assessed physical home environment, facilities, and financial resources. We found this was associated with depression, but primarily at the deprived end of the distribution, and in men. In particular, among men experiencing poor standards of living those who were under-employed were most likely to suffer from depression. Furthermore, living in a more heavily urbanised area was associated with depression in men alone. Although these relationships persisted after controlling for socio-demographic factors, at least some of these associations may be an outcome rather than a cause of depression, because the data are cross-sectional.

We further explored specific components of poor standards of living. The risk factors found were mainly objective (e.g., structural materials of the housing unit, as observed by interviewers) but one component assessed how well the participant felt he or she was managing financially, which could have been influenced by negative reporting biases according to depressed mood. Poverty and low income have been associated with depression in many settings (Patel et al., 1999, 2006). We found that basic facilities had the strongest relationship to depression, rather than factors such as car or house ownership which have been found to be important in more developed countries such as the UK (Lewis et al., 2003). This may indicate the importance of material standards of living relative to other people in one's own country (rather than absolute levels). Alternatively, this may be due to differing power to detect associations according to the frequency of exposure.

Poor quality structural materials, poor quality of toilet or water facilities and recent hunger due to poverty were only associated with depression in men. Interestingly, this corresponds with genetic analyses on the same sample, which suggested an overall greater influence of environments (and a lesser proportional influence of genes) in men compared to women (Ball et al., 2009). In Sri Lanka men are primarily responsible for the quality of housing, either because they build the house, or through their wages. Their failure to provide would be evident when their housing or finances are in an obviously poor state, and when they are not employed as often as they are able. In contrast, among women of low standards of living, those who worked full-time were more susceptible to depression than those who did not work. Work may be a mark of high status in women who are relatively well-off, but a mark of low status and an indicator of stress where it is a material necessity. Men who are unable to find full employment and women who are required to work may also lack social support. In addition, living in an urban area was a risk factor for depression in men only, which might be a reflection of different social or economic pressures on men in more heavily urbanised areas.

### 4.4 Limitations

Research that attempts a population-comparative approach to assessing levels of psychiatric disorders assumes that the same construct can be detected more or less well in people from multiple populations or contexts. Measurement in the current study was based on the Western-derived DSM model of depression, and as a consequence it may have missed Sri Lanka-specific manifestations. The results gave no evidence to support different underlying psychopathology of depression in Sri Lanka (the symptom profile and socio-demographic and environmental correlates were similar) except for a lower prevalence.

This sample is broadly representative of people living in the Colombo District of Sri Lanka, although ethnic minorities were slightly under-represented. Regions outside of Colombo District are more rural, and more people in other specific areas would have been more heavily involved in the civil war and experiences of the tsunami.

## 5 Conclusion

In conclusion, the characteristics of depression appear very similar in this area of Sri Lanka to the rest of the world. Differences in environmental exposures and the cultural context may contribute to different aetiological pathways to depression compared to Western or more developed countries. Further research will help uncover these mechanisms, and improve our understanding of depression both in Sri Lanka and the wider world. In particular, the genetically informative design of the CoTASS sample could help tease apart the causal connections between environmental exposures and depressive outcomes.

## Role of the funding source

The Wellcome Trust (Grant Number 069629) provided funding for the CoTASS study, and had no further role in study design; in the collection, analysis and interpretation of data; in the writing of the report; and in the decision to submit the paper for publication. The Institute for Research and Development, Sri Lanka, provided infrastructural support.

## Conflict of interest

PM has received honoraria from Eli Lilly and GSK and has acted as a consultant in the recent past for GSK and Astra Zeneca. NG has received honoraria from Sanofi-Aventis and Servier. All other authors declare that they have no potential conflicts of interest.

## Figures and Tables

**Table 1:** The prevalence of depression among different socio-demographic groups, according to different definitions of disorder.

	DSM, current n/N (%)	DSM, past year n/N (%)	DSM, lifetime n/N (%)	DSM excluding impairment, lifetime n/N (%)	D-probe, lifetime n/N (%)	DSM excluding impairment, lifetime Adjusted OR^a^
*Whole sample*	94/5937 (1.58)	159/5937 (2.68)	395/5973 (6.61)	667/5973 (11.17)	945/5973 (15.82)	–
(95% CI)	(1.27–1.90)	(2.27–3.09)	(5.98–7.24)	(10.37–11.97)	(14.90–16.75)	–

*Twin status*						
Non-twin	40/2005 (2.00)	56/2005 (2.79)	134/2018 (6.64)	218/2018 (10.80)	317/2018 (15.71)	–
Twin	54/3932 (1.37)^	103/3932 (2.62)	261/3995 (6.60)	449/3995 (11.35)	628/3995 (15.88)	0.83 (0.69–1.01)

*Sex*						
Male	37/2737 (1.35)	54/2737 (1.97)	132/2745 (4.81)	219/2745 (7.98)	368/2745 (13.41)	–
Female	57/3198 (1.78)	105/3198 (3.28)**	262/3226 (8.12)**	447/3226 (13.86)**	576/3226 (17.85)**	1.67 (1.39–2.01)

*Age (quartiles)*						
1st	13/1443 (0.90)	29/1443 (2.01)	60/1449 (4.14)	116/1449 (8.01)	170/1449 (11.73)	–
2nd	25/1492 (1.68)^	42/1492 (2.82)	105/1504 (6.98)**	165/1504 (10.97)*	243/1504 (16.16)**	1.75 (1.22–2.51)
3rd	28/1422 (1.97)*	50/1422 (3.52)*	115/1430 (8.04)**	188/1430 (13.15)**	261/1430 (18.25)**	2.87 (1.77–4.68)
4th	28/1575 (1.78)*	38/1575 (2.41)	114/1585 (7.19)**	197/1585 (12.43)**	270/1585 (17.03)**	3.94 (1.93–8.07)

*Ethnicity*						
Sinhala	89/5483 (1.62)	149/5483 (2.72)	374/5517 (6.78)	635/5517 (11.51)	902/5517 (16.35)	–
Non-Sinhala	5/454 (1.10)	10/454 (2.20)	21/456 (4.61)^	32/456 (7.02)*	43/456 (9.43)**	0.53 (0.35–0.82)

*Marital status*						
Married	59/3542 (1.67)	91/3542 (2.57)	232/3566 (6.51)	395/3566 (11.08)	566/3566 (15.87)	–
Never married	22/2016 (1.09)^	47/2016 (2.33)	105/2028 (5.18)*	179/2028 (8.83)*	266/2028 (13.12)**	0.91 (0.70–1.19)
Previously married	13/373 (3.49)*	21/373 (5.63)**	57/373 (15.28)**	92/373 (24.66)**	112/373 (30.03)**	2.73 (2.02–3.71)

*Years of schooling*						
Up to 10	49/2157 (2.27)	75/2157 (3.48)	164/2171 (7.55)	284/2171 (13.08)	395/2171 (18.19)	–
11–12	29/2023 (1.43)*	52/2023 (2.57)^	131/2036 (6.43)	207/2036 (10.17)**	295/2036 (14.49)**	0.82 (0.67–1.01)
13+	15/1712 (0.88)**	31/1712 (1.81)**	97/1721 (5.64)*	170/1721 (9.88)**	244/1721 (14.18)**	0.78 (0.63–0.97)

*Urbanicity*						
Semi-urban	50/3603 (1.39)	94/3603 (2.61)	243/3629 (6.70)	409/3629 (11.27)	588/3629 (16.20)	–
Urban	44/2332 (1.89)	65/2332 (2.79)	152/2342 (6.49)	258/2342 (11.02)	357/2342 (15.24)	
(Male)						1.41 (1.03–1.93)
(Female)						0.91 (0.69–1.10)

Asterisks show unadjusted association: *P < 0.05 **P < 0.01 ^*P* < 0.10. Previously married = widowed/separated/divorced.

a Adjusted for all variables in this table (except urbanicity), plus age as a continuous variable.

**Table 2:** Endorsement of symptom items among depressed participants, according to different definitions of disorder.

	Symptom	DSM, current (%)	DSM, past year (%)	DSM, lifetime (%)	DSM excluding impairment, lifetime (%)	D-probe, lifetime (%)
		*N* = 94	*N* = 155	*N* = 395	*N* = 667	*N* = 945
1	Sad mood	99	95	94	92	86
2	Loss of interest	95	92	91	90	81
3a	Decreased appetite	80	82	85	83	65
3b	Decreased weight	65	58	59	54	41
3c	Increased appetite	6	6	5	4	3
3d	Increased weight	6	4	4	2	2
4a	Insomnia	86	87	86	85	71
4b	Hypersomnia	36	30	26	21	18
5a	Psychomotor retardation	58	62	64	59	46
5b	Agitation	54	57	53	46	35
6	Lack of energy/feel tired	84	82	80	76	56
7	Guilt/worthlessness	16	19	20	18	14
8	Diminished concentration/thinking/deciding	96	96	97	97	81
9	Thoughts of death	69	69	59	53	41
Average number endorsed (max = 9)⁎		7.1	7.1	7.0	6.7	5.6

⁎A symptom is counted as present if a subject responds positively to at least one corresponding item, giving a maximum score of 9 symptoms from 14 items.

**Table 3:** The relationship between depression (‘DSM excluding impairment’ definition) and standard of living.

Standard of living variable	n/N (depressed/exposed)	Unadjusted OR	Adjusted OR^a^	Moderation by sex *z*-score (*P*)^a^	PAF
*SoL (standardised)*	–	1.19 (1.10–1.28)	1.16 (1.06–1.27)	− 1.97 (0.05)	
M	–	1.28 (1.14–1.45)	1.32 (1.16–1.50)		
F	–	1.13 (1.02–1.25)	1.07 (0.96–1.20)		

*SoL quintiles*					
Top 3 quintiles	359/3583	–	–	–	–
Bottom 2 quintiles	307/2385	1.33 (1.12–1.57)	1.25 (1.05–1.49)	− 2.32 (0.02)	0.11
M	110/1065	1.66 (1.25–2.20)	1.76 (1.30–2.36)		0.20
F	197/1320	1.16 (0.94–1.43)	1.05 (0.84–1.30)		–

*Environmental risk components*					
Informal house tenure/type	72/611	1.07 (0.82–1.39)	1.09 (0.81–1.46)	0.01 (0.99)	0.01
Overcrowding	95/674	1.39 (1.10–1.76)	1.27 (0.99–1.64)	− 0.56 (0.58)	0.04
Poor quality structural materials	81/629	1.20 (0.93–1.55)	1.08 (0.83–1.41)	− 2.05 (0.04)	0.02
M	33/285	1.60 (1.07–2.40)	1.61 (1.06–2.43)		0.06
F	48/344	1.01 (0.72–1.41)	0.85 (0.60–1.19)		< 0.01
Low quality toilet/water	104/742	1.36 (1.07–1.72)	1.29 (1.01–1.65)	− 2.59 (0.01)	0.04
M	42/322	1.90 (1.33–2.72)	1.94 (1.34–2.81)		0.09
F	62/420	1.09 (0.81–1.48)	1.01 (0.74–1.37)		0.01
Low quality lighting/fuel	350/3078	1.05 (0.88–1.24)	0.94 (0.79–1.13)	− 1.29 (0.20)	0.02
Lack fridge/phone	164/1237	1.29 (1.06–1.56)	1.12 (0.92–1.38)	0.08 (0.93)	0.05
Lack transport	397/3337	1.18 (1.00–1.39)	1.08 (0.90–1.28)	1.15 (0.25)	0.09
Hunger due to lack of money	50/229	2.32 (1.68–3.21)	2.05 (1.44–2.91)	− 3.26 (< 0.01)	0.04
M	22/82	4.58 (2.75–7.62)	4.22 (2.41–7.40)		0.08
F	28/147	1.49 (0.97–2.30)	1.37 (0.87–2.16)		0.02
Subjective report of financial difficulty	135/777	1.84 (1.49–2.28)	1.69 (1.35–2.12)	− 1.06 (0.29)	0.09

SoL: Standard of living composite scale.

a Adjusted for demographic factors: sex, age (as continuous variable), ethnicity, previously married status (i.e., widowed, separated or divorced), less than 10 years at school, urbanicity. M: males F: females PAF: Population attributable fraction.

**Table 4:** The influence of marital and occupational status on depression, stratified by standard of living.

	Bottom 40% of SoL		Top 60% of SoL	
	n/N (depressed/exposed) (%)	Adjusted OR^a^	n/N (depressed/exposed) (%)	Adjusted OR^a^
*Men*	110/1065 (10.6)		109/1678 (6.5)	
Married	71/661 (10.3)	–	65/948 (6.9)	–
Never married	29/378 (7.7)	0.59 (0.33–1.05)	40/711 (5.6)	0.77 (0.44–1.35)
W/S/D	10/26 (38.5)	5.35 (2.27–12.59)	4/19 (21.1)	3.71 (1.17–11.76)
Work 11/12mths	58/586 (9.9)	–	69/1038 (6.6)	–
Worked 1–10mths	32/195 (16.4)	1.71 (1.07–2.75)	13/157 (8.3)	1.24 (0.66–2.33)
Worked 0 mth	19/225 (8.4)	0.74 (0.42–1.30)	26/410 (6.3)	0.92 (0.55–1.54)

*Women*	197/1320 (14.9)		250/1905 (13.1)	
Married	115/799 (14.4)	–	144/1158 (12.4)	–
Never married	39/352 (11.1)	0.79 (0.51–1.20)	71/587 (12.1)	0.84 (0.58–1.22)
W/S/D	43/169 (25.4)	1.92 (1.19–3.11)	35/159 (22.0)	2.29 (1.36–3.84)
Work 11/12	62/306 (20.3)	–	69/539 (12.8)	–
Work 1–10	27/159 (17.0)	0.79 (0.48–1.30)	28/152 (18.4)	1.56 (0.94–2.57)
Work 0 mth	107/804 (13.3)	0.59 (0.42–0.82)	149/1170 (12.7)	0.95 (0.69–1.31)

SoL: Standard of living composite scale.

a Adjusted for demographic factors: sex, age (as continuous variable), ethnicity, previously married status, less than 10 years at school, urbanicity. W/S/D = widowed, separated or divorced. Work = number of months worked in the past 12 months.
